# Phenolic contents, antioxidant and anticholinesterase potentials of crude extract, subsequent fractions and crude saponins from Polygonum hydropiper L

**DOI:** 10.1186/1472-6882-14-145

**Published:** 2014-05-03

**Authors:** Muhammad Ayaz, Muhammad Junaid, Jawad Ahmed, Farhat Ullah, Abdul Sadiq, Sajjad Ahmad, Muhammad Imran

**Affiliations:** 1 Department of Pharmacy, University of Malakand, Khyber Pakhtoonkhwa 18000, KPK, Pakistan; 2 Institute of Basic Medical Sciences (IBMS), Khyber Medical University (KMU), Peshawar, Pakistan

**Keywords:** DPPH, ABTS, Gallic acid, *Polygonum hydropiper* L

## Abstract

**Background:**

We investigated *Polygonum hydropiper* L. (*P. hydropiper*) for phenolic contents, antioxidant, anticholinesterase activities, in an attempt to rationalize its use in neurological disorders.

**Methods:**

Plant crude extract (Ph.Cr), its subsequent fractions: *n*-hexane (Ph.Hex), chloroform (Ph.Chf), ethyl acetate (Ph.EtAc), *n*-Butanol (Ph.Bt), aqueous (Ph.Aq) and saponins (Ph.Sp) were evaluated for 1,1-diphenyl,2-picrylhydrazyl (DPPH), 2,2-azinobis[3-ethylbenzthiazoline]-6-sulfonic acid (ABTS) free radical scavenging potential. Further, acetylcholinesterase (AChE) & butyrylcholinesterase (BChE) inhibitory activities were performed using Ellman's assay. Moreover, total phenolic contents of plant extracts were determined and expressed in mg of gallic acid equivalent per gram of dry sample (mg GAE/g dry weight).

**Results:**

Among different fractions, Ph.Cr (90.82), Ph.Chf (178.16), Ph.EtAc (203.44) and Ph.Bt (153.61) exhibited high phenolic contents. All fractions showed concentration dependent DPPH scavenging activity, with Ph.EtAc 71.33% (IC_50_ 15 μg/ml), Ph.Bt 71.40% (IC_50_ 3 μg/ml) and Ph.Sp 71.40% (IC_50_ 35 μg/ml) were most potent. The plant extracts exhibited high ABTS scavenging ability i.e. Ph.Bt (91.03%), Ph.EtAc (90.56%), Ph.Sp (90.84%), Ph.Aq (90.56%) with IC_50_ < 0.01 μg/ml. All fractions showed moderate to high AChE inhibitory activity as; Ph.Cr, 86.87% (IC_50_ 330 μg/ml), Ph.Hex, 87.49% (IC_50_ 35 μg/ml), Ph.Chf, 84.76% (IC_50_ 55 μg/ml), Ph.Sp, 87.58% (IC_50_ 108 μg/ml) and Ph.EtAc 79.95% (IC_50_ 310 μg/ml) at 1 mg/ml). Furthermore the BChE inhibitory activity was most prominent in Ph.Hex 90.30% (IC_50_ 40 μg/ml), Ph.Chf 85.94% (IC_50_ 215 μg/ml), Ph.Aq 87.62% (IC_50_ 3 μg/ml) and Ph.EtAc 81.01% (IC_50_ 395 μg/ml) fractions.

**Conclusions:**

In this study, for the first time, we determined phenolic contents, isolated crude saponins, investigated antioxidant and anticholinestrase potential of *P. hydropiper* extracts. The results indicate that *P. hydropiper* is enriched with potent bioactive compounds and warrant further investigation by isolation and structural elucidation to find novel and affordable compounds for the treatment of various neurological disorders.

## Background

Medicinal plants are nature’s gift and are necessary for disease-free & healthy life of human beings. Since ancient times, plants have been employed for prevention and treatment of a variety of ailments. There has been a great revival in the use of herbal remedies particularly in last decades. According to World Health Organization (WHO), approximately eighty percent of the world population relies on medicinal plants to meet their primary health care [1]. *P. hydropiper,* also called Smartweed (family *polygonaceae*), has a long history of herbal use, equally in Eastern & Western herbal medicine. Domestically, it is used as anti-inflammatory, carminative, astringent, diuretic, CNS stimulant, diaphoretic, stomachic, styptic, in bleeding and in diarrhea [2]. The plant is enriched with rutin which strengthens fragile blood vessels and helps in the prevention of bleeding [3]. Traditionally, the whole plant decoction is used to treat an extensive range of ailments like dyspepsia, diarrhea, menorrhagia, hemorrhoids and skin itching [4]. Recently antioxidant flavonoids have been isolated from leaves of *P. hydropiper *[5]. Other species of polygonaceae family have been reported for their effectiveness in cerebral ischemia [6], parkinson's disease [7] and neuroprotective effects [8].

Free radicals are implicated in the progression of a variety of disorders in humans including central nervous system injury, arthritis, atherosclerosis, ischemic heart diseases, gastritis, cancer and reperfusion injury of many tissues [9,10]. Free radicals from environmental pollutants, chemical agents, radiations, toxins, spicy and deep fried foods cause reduction of immune system antioxidants, alter gene expression along with induction of abnormal proteins. Free radicals are generated in living systems during oxidation process. To counteract oxidative stress, catalase and hydroperoxidase enzymes in human body convert hydrogen peroxide and hydroperoxides to nonradical forms and hence work as natural antioxidants. Thus, due to depletion of human immune system, natural antioxidants as free radical scavengers may be necessary [11,12]. Presently available synthetic antioxidants including butylated hydroxy toluene (BHT), butylated hydroxy anisole (BHA), gallic acid esters and tertiary butylated hydroquinon have been alleged to be associated with negative health consequences. Hence, their use is restricted and there is a tendency to substitute them with natural antioxidants [13]. Numerous reports on the antioxidant and radical-scavenging activities of crude extracts and pure natural compounds have been published [10,14]. Among the antioxidant compounds, significant attention has been given to phenolic compounds and flavonoids. Phenolic compounds, due to the existence of the conjugated ring structures and hydroxyl groups, have the potential to function as antioxidants by scavenging the free radicals that are involved in oxidative processes via hydrogenation or complexation with oxidizing species [15].

Acetylcholinesterase (AChE) and butyrylesterase (BChE) are useful targets for the development of novel and mechanism based inhibitors, due to its role in the breakdown of acetylcholine (ACh) neurotransmitter. Inhibitors of AChE and BChE enzymes are the most valuable approaches to treat neurological diseases including Alzheimer's disease (AD) [16] and possible beneficial applications in the treatment of Parkinson's disease, ataxia and dementia [17]. AD is a persistent neurological disorder frequently associated with memory impairment, behavioral turbulence, cognitive dysfunction and imperfection in routine life activities [18,19]. This disease results from malfunctioning of different biochemical pathways resulting in a significant decline in ACh amount [20]. ACh is involved in signal transmission in the synapse and its pharmacological action is terminated primarily by AChE and secondly by BChE [21]. Therefore inhibitors of these metabolizing enzymes have become the important alternatives in treatment of AD and other neurological diseases. Conversely, presently available drugs are associated with serious side effects including hepatotoxicity [22] and are only effective in mild type of AD [23]. Consequently, it is necessary to embark new, safe and effective drug candidates. Plants are potential sources of novel active compounds and have a long history of therapeutic use since the beginning of human era. Galanthamine, an anticholinestrase alkaloid isolated from snowdrop, has been recently approved for the treatment of AD [24]. Research has been focused on the biological effects of plants which have been traditionally used as cholinesterase inhibitors *in-vitro* as well as *in-vivo *[24,25]. This study is focused on preliminary anticholinestrase and antioxidant potential of *P. hydropiper*.

## Methods

### Plant Collection, Extraction and Fractionation

*P. hydropiper* whole plant was collected from Talash Valley, Khyber Pakhtoonkhwa, Pakistan in July, 2013. The plant was identified by botanical taxonomist at Arid agriculture University, Rawalpindi, Pakistan and a sample was deposited at the herbarium, University of Malakand Chakdara (Dir), Pakistan with voucher no (H.UOM.BG.107). Plant material was cleansed with distilled water and was shade dried for 15 days. Thereafter, it was coarsely crushed using cutter mill. The powder material (4.5 kg) was soaked in 80% methanol (22 L) for 10 days with frequent shaking. Extraction with methanol was repeated three times, added to original extract and filtered through muslin cloth and then through filter [26]. The filtrate was concentrated using rotary evaporator (Heidolph Laborota 4000, Schwabach, Germany) under reduced pressure at 40°C resulting in 290 g (6.44%) of dark brown colored semisolid mass. Crude methanolic extract (250 g) of *P. hydropiper* (Ph-Cr) was suspended in 500 ml of distilled water and consequently partitioned with *n*-hexane (3 × 500 ml), chloroform (3 × 500 ml), ethyl acetate (3 × 500 ml), *n*-butanol (3 × 500 ml) and aqueous (3 × 500 ml), using separating funnels. Finally, 68g (27.2%) Ph.Hex, 27g (10.8%) Ph.Chf, 13g (5.2%) Ph.EtAc, 11 g (4.4%) Ph.Bt & 37 g (14.8%) Ph.Aq fractions were obtained.

### Extraction of crude saponins

A portion of powdered plant material, weighing 60 g, was transferred to a conical flask and was soaked with 100 ml of 20% ethanol. The mixture was heated for 4 h at a temperature of 55°C using water bath and constant shaking. Thereafter, it was filtered and was again extracted with 200 ml of 20% ethanol. Volume of the liquid extract was reduced to 40 ml using water bath and transferred it to a separating funnel. Diethyl ether (20 ml) was added to it with vigorous shaking until two layers were formed. Organic layer was discarded, whereas 60 ml of *n*-butanol was added to aqueous fraction. The combined aqueous-butanol mixture was washed with 5% NaCl solution two times. Finally solvents were evaporated using water bath to get saponins (9 g) with a percent yield of 15% [27].

### Chemical and Drugs

DPPH (CAS 1898-66-4 Sigma Aldrich CHEMIE GmbH USA), ABTS (CAS 30931-67-0 Sigma Aldrich USA), K_2_S_2_O_4_ (Riedel-de Haen Germany), Gallic acid (CAS 149-91-7 GmbH USA) and Folin Ciocalteu reagent (FCR) was purchased from Merck Co. (Germany). Enzymes including AChE *Electric eel* (type-VI-S, CAS 9000-81-1 Sigma-Aldrich GmbH USA), BChE equine serum Lyophilized (CAS 9001-08-5 Sigma-Aldrich GmbH USA), substrates acetylthiocholine iodide (CAS1866-15-5 Sigma-Aldrich UK), butyrylthiocholine Iodide CAS 2494-56-6 Sigma-Aldrich Switzerland), DTNB 5,5-dithio-bis-nitrobenzoic acid (CAS 69-78-3 Sigma-Aldrich Germany), Galanthamine hydrobromide Lycoris Sp. (CAS 1953-04-4 Sigma-Aldrich France) were used for enzyme inhibition study. For preparation of buffer, di-potassium hydrogen phosphate (K_2_HPO_4_), Potassium di-hydrogen phosphate (KH2PO_4_), potassium hydroxide used were of extra pure analytical grade.

### Total phenolic contents

Total phenolic contents of the fractions were investigated using procedure adopted by Kim *et al., *[28]. One ml from each concentration of the plant extract was added to 9 ml distilled water followed by addition of 1 ml FCR with vigorous shaking. After five minutes, 10 ml of 7% Na_2_CO_3_ solution was added to the tube and mixed properly. Distilled water (25 ml) was added to this mixture and analyzed after 90 minutes using spectrophotometer (Thermo electron corporation, USA) at 750 nm. Finally gallic acid (97.5% pure) standard curve was employed to quantify total phenolic contents and were expressed as mg equivalent of gallic acid.

### DPPH radical scavenging assay

Free radical scavenging ability of the samples, based on the scavenging activity of 1,1-diphenyl,2-picrylhydrazyl (DPPH) free radical, was evaluated using the procedure described previously [29]. Different dilutions (125, 250, 500 and 1000 μg/ml) of plant extract (0.1 ml) were added to 0.004% methanolic solution of DPPH. After 30 minutes, absorbance was determined at 517 nm using UV spectrophotometer. Ascorbic acid was used as positive control, percent scavenging activity was calculated as; [(A_0_ - A_1_)/A_0_] × 100, where A_0_ represent absorbance of control and A_1_ is the absorbance of the plant extracts. Each experiment was done in triplicate and inhibition graphs were constructed using the GraphPad prism program (GraphPAD, San Diego, California, USA) and median inhibitory concentrations IC_50_ values were determined.

### ABTS free radical scavenging assay

The antioxidant potential of *P. hydropiper* was also evaluated using 2, 2-azinobis [3-ethylbenzthiazoline]-6-sulfonic acid (ABTS) [30]. The assay is based on the capacity of antioxidants to scavenge ABTS radical cation causing a reduction in absorbance at 734 nm. briefly, ABTS 7 mM and potassium persulphate (K_2_S_2_O_4_) 2.45 mM solutions were prepared and mixed. The resultant mixture was stored in dark at room temperature for 12-16 h to get dark colored solution containing ABTS radical cations. Prior to use, ABTS radical cation solution was diluted with Phosphate buffer (0.01 M) pH 7.4, to adjust an absorbance value of 0.70 at 734 nm. Radical scavenging ability of the fractions was analyzed by mixing 300 μl of test sample with 3.0 ml of ABTS solution in cuvette. The reduction in absorbance was measured spectrophotometrically after one minute of mixing the solutions and continued for six min. Ascorbic acid was used as positive control. The assay was repeated in triplicate and percentage inhibition was calculated using formula:

$$\begin{array}{l} \%\;\text{scavenging}\;\text{effect}=\text{control}\;\text{absorbance}\\ \;-\;\text{sample}\text{absorbance}\\ \;/\text{control}\;\text{absorbance}\times 100 \end{array}$$

The antioxidant effect was expressed in terms of percent inhibition and as EC_50_ (Extract concentration required for 50% reduction of ABTS radicals).

### Anticholinesterase assays

AChE from *Electric eel* and BChE from equine serum were used to explore the enzymes inhibitory potential of Ph.Cr of *P. hydropiper*, its subsequent fractions and Ph.Sp using Ellman's assay [31,32]. The assay is based on the hydrolysis of acetylthiocholine iodide or butyrylthiocholine iodide by the respective enzymes and the formation of 5-thio-2-nitrobenzoate anion followed by complexation with DTNB to give yellow color compound which is detected with spectrophotometer beside the reaction time.

#### *Preparation of solutions*

Ph.Cr and subsequent fractions were dissolved in phosphate buffer (0.1 M) in concentrations ranging from 125-1000 μg/ml. For the preparation of 0.1 M and 8.0 ± 0.1 pH phosphate buffer solution, K_2_HPO_4_ (17.4 g/L) and KH2PO_4_ (13.6 g/L) were prepared and were mixed in 94% and 6% ratio respectively. Finally, potassium hydroxide was used to adjust PH. AChE (518U/mg solid) and BChE (7-16U/mg) were diluted in freshly prepared buffer pH 8.0 until final concentrations of 0.03U/ml and 0.01U/ml were obtained. Solutions of DTNB (0.0002273 M), ATchI and BTchI (0.0005 M) were prepared in distilled water and were kept in eppendorf caps in the refrigerator at 8°C. Galanthamine (Positive control) was dissolved in methanol and the aforementioned dilutions were prepared.

#### *Spectroscopic analysis*

For each assay, an enzyme solution of 5 μl was added to the cuvette followed by addition of plant extract solution (205 μl), and finally DTNB reagent (5 μl). The solution mixture was maintained at 30°C for 15 min using water bath with subsequent addition of substrate solution (5 μl) was added. A double beam spectrophotometer (Thermo electron corporation USA) was used to measure the absorbance at 412 nm. Negative control contained all components apart from the plant extracts, whereas positive control galanthamine (10 μg/ml) was used in the assay as standard cholinesterase inhibitor. The absorbances along with the reaction time were taken for four minutes at 30°C. The experiments were performed in triplicate. The enzyme activity and enzyme inhibition by control and tested samples were calculated from the rate of absorption with change in time (V = ΔAbs /Δt) as follow;

Enzyme inhibition (%) = 100 - percent enzyme activity

Enzyme activity (%) = 100 × V/Vmax where (Vmax) is enzyme activity in the absence of inhibitor drug.

### Estimation of IC_50_ values

Concentrations of the plant extracts which inhibited substrate hydrolysis (AChE and BChE) by 50% (IC_50_). Radical scavenging ability was calculated by a linear regression analysis among the percent inhibition against the extract concentrations via Excel program.

### Statistical data analysis

All the assays were repeated in triplicate and vales were expressed as means ± S*.*E*.*M. Significance between antioxidant activity and plant extracts were analyzed using Mann–Whitney U test. Group comparison was done using Student’s *t*-test. The P values less than 0.05 were considered as statistically significant.

## Results

### Total phenolic content

**Figure 1:**
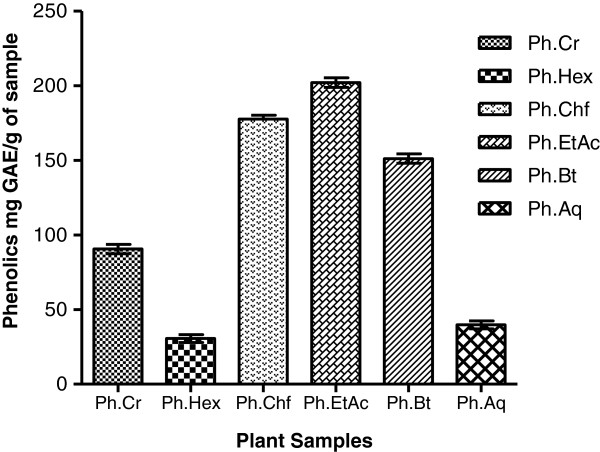
**Total phenolic content in different fractions of *P. hydropiper*.** Activity expressed as mg GAE/g of sample (mean ± SEM n = 3).

The extraction yield of phenolics (mg GAE/g of sample) in different fractions of the plant extracts are summarized in Figure 1. Ph.EtAc and Ph.Chf exhibited high phenolic contents as compared to other fractions. The concentration of phenolics among different fractions were in an ascending order of Ph.EtAc > Ph.Chf > Ph.Bt > Ph.Cr > Ph.Aq > Ph.Hex.

### DPPH Free radical scavenging potential

**Figure 2:**
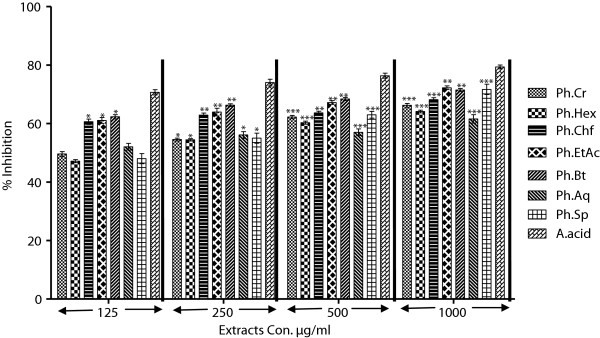
**Antioxidant assay of plant extracts using DPPH assay.** Values represent% radical scavenging (mean ± SEM) of three replicates. Values significantly different as compare to positive control, *P < 0.05, **P < 0.01, ***P < 0.001.

In the DPPH free radical scavenging assay, all fractions showed concentration dependent inhibition of the free radicals as shown in Figure 2. Among different fractions, Ph.EtAc, Ph.Bt and Ph.Chf showed highest activity which can be attributed to their high phenolic contents. Ph.Bt, Ph.Chf and Ph.EtAc fractions were most potent with IC_50_ of 3, 10 and 15 μg/ml respectively. The DPPH free radical scavenging potential of the tested fractions were in an ascending order of Ph.EtAc > Ph.Bt > Ph.Chf > Ph.Sp > Ph.Cr > Ph.Hex > Ph.Aq. In comparison to positive control, Ph.Cr, Ph.Hex, Ph.Chf, Ph.Aq and Ph.Sp percent inhibitions were significantly different P < 0.001 at highest concentration of extracts (1000 μg/ml).

### ABTS Free radical scavenging assay

Results of ABTS free radical scavenging assay are given in Table 1. Plant extracts revealed high ABTS free radical scavenging activity in comparison to DPPH. Among different fractions, Ph.Bt, Ph.EtAc, Ph.Aq, Ph.Sp revealed highest ABTS scavenging activity causing 91.03, 90.65, 90.56 and 90.84% inhibition of free radicals respectively. The scavenging activity of these fractions were comparable with the positive control ascorbic acid with IC_50_ values of 0.01 μg/ml.

**Table 1:** ABTS free Radical scavenging assay of plant extracts using ascorbic acid as standard

**Samples**	**Concentrations (μg/ml)**	**Percent inhibition (mean ± SEM)**	**IC**_**50 **_**(μg/ml)**
Ph.Cr	1000	88.58 ± 1.12	< 0.01
	500	87.65 ± 1.34	
	250	84.31 ± 2.15	
	125	77.56 ± 1.73*	
	1000	88.44 ± 0.58	
	500	87.90 ± 0.96	
Ph.Hex	250	86.28 ± 2.19	< 0.01
	125	81.77 ± 1.24	
	1000	89.76 ± 0.71	
	500	87.65 ± 1.32	
Ph.Chf	250	84.23 ± 1.83	< 0.01
	125	79.10 ± 0.90*	
	1000	90.56 ± 1.06	
	500	87.42 ± 0.43	
Ph.EtAc	250	86.42 ± 0.46	< 0.01
	125	80.90 ± 1.55	
	1000	91.03 ± 0.35	
	500	90.08 ± 0.47	
Ph.Bt	250	87.91 ± 0.88	< 0.01
	125	83.80 ± 1.50	
	1000	90.56 ± 1.06	
	500	90.08 ± 0.47	
Ph.Aq	250	87.91 ± 0.88	< 0.01
	125	83.80 ± 1.50	
	1000	90.84 ± 0.30	
	500	88.72 ± 1.01	
Ph.Sp	250	87.94 ± 1.13	< 0.01
	125	79.80 ± 0.90*	
	1000	87.90 ± 0.96	
	500	83.08 ± 0.47	
Ascorbic acid	250	79.85 ± 2.24	< 0.1
	125	77.40 ± 0.20	

Values significantly different as compare to positive control, *P < 0.05.

Values expressed as Percent inhibition (Mean ± SEM of n = 3) and IC_50_.

### Acetylcholinesterase Inhibition assay

Among different fractions of *P. hydropiper,* Ph.Sp, Ph.Hex and Ph.Cr fractions showed strongest activity causing 87.58, 87.49 and 86.87% inhibition of AChE. All fractions were effective in concentration dependent manner as summarized in Table 2. Ph.Hex , Ph.Chf and Ph.Sp were most potent displaying median inhibitory values (IC_50_) of 35, 55 and 100 μg/ml. Whereas the IC_50_ value for positive control galanthamine was 0.1 μg/ml. The AChE inhibitory activity of the tested fractions were in an ascending order of Ph.Sp > Ph.Hex > Ph.Cr > Ph.Chf > Ph.EtAc > Ph.Bt > Ph.Aq.

**Table 2:** AChE inhibitory potential of plant extracts

**Samples**	**Concentrations (μg/ml)**	**Percent inhibition (mean ± SEM)**	**IC**_**50 **_**(μg/ml)**
Ph.Cr	1000	86.87 ± 1.27	330
	500	80.62 ± 1.67**	
	250	31.31 ± 0.58***	
	125	27. 22 ± 1.28***	
Ph.Hex	1000	87.49 ± 0.60	35
	500	76.28 ± 1.94**	
	250	70.08 ± 1.04***	
	125	65.37 ± 0.56***	
Ph.Chf	1000	84.76 ± 0.61	55
	500	81.36 ± 1.31**	
	250	66.27 ± 1.06***	
	125	61.17 ± 1.30***	
Ph.EtAc	1000	79.95 ± 2.01**	310
	500	58.89 ± 4.82***	
	250	46.22 ± 1.28***	
	125	40.51 ± 0.54***	
Ph.Bt	1000	75.52 ± 3.28**	240
	500	55.59 ± 3.28***	
	250	50.83 ± 1.21***	
	125	45.87 ± 0.85***	
Ph.Aq	1000	67.60 ± 1.63***	100
	500	64.42 ± 1.89***	
	250	58.25 ± 1.40***	
	125	51.10 ± 0.60***	
Ph.Sp	1000	87.58 ± 0.63	108
	500	86.61 ± 0.43	
	250	60.93 ± 0.67***	
	125	53.65 ± 0.91***	
	1000	95.83 ± 1.21	
Galanthamine	500	93.58 ± 0.63	< 0.1
	250	87.45 ± 0.90	
	125	83.08 ± 1.04	

Result expressed as% inhibition (mean ± SEM of n = 3) and IC_50_ values. Values significantly different as compare to positive control, *:P < 0.05, **:P < 0.01, ***:P < 0.001.

### Butyrylcholinesterase inhibition assay

BChE inhibitory activity was most prominent for, Ph.Hex, Ph.Aq, Ph.Chf and Ph.EtAc fractions at 1 mg/ml concentration. Results are given in Table 3. Ph.Hex, Ph.Aq and Ph.Chf exhibited 90.30, 87.62 and 85.94% inhibition of BChE which is comparable to positive cotrol galantamine causing 96% enzyme inhibition at the same concentration (1 mg/ml). Whereas Ph.Aq and Ph.Hex were more potent with IC_50_ values of 3 and 40 μg/ml.

**Table 3:** BChE inhibitory potential of plant extracts

**Samples**	**Concentrations (μg/ml)**	**Percent inhibition (mean ± SEM)**	**IC**_**50 **_**(μg/ml)**
Ph.Cr	1000	75.29 ± 0.64**	285
	500	64.72 ± 0.89***	
	250	47.44 ± 0.86***	
	125	42.78 ± 0.45***	
Ph.Hex	1000	90.30 ± 1.42	40
	500	84.80 ± 0.41*	
	250	69.51 ± 0.59***	
	125	65.90 ± 0.32***	
Ph.Chf	1000	85.94 ± 0.91*	215
	500	62.93 ± 1.73***	
	250	51.82 ± 0.95***	
	125	46.68 ± 0.22***	
Ph.EtAc	1000	81.01 ± 0.97**	395
	500	59.05 ± 1.03***	
	250	34.54 ± 0.60***	
	125	29.88 ± 0.89***	
Ph.Bt	1000	76.92 ± 1.15**	415
	500	58.89 ± 1.73***	
	250	21.65 ± 2.41***	
	125	18. 20 ± 0.47***	
Ph.Aq	1000	87.62 ± 1.42	3
	500	84.79 ± 1.88	
	250	80.79 ± 1.08*	
	125	75.12 ± 0. 54**	
Ph. Sp	1000	76.32 ± 0.87**	330
	500	74.33 ± 0.66**	
	250	35.40 ± 0.82***	
	125	30. 90 ± 0.45***	
Galantamine	1000	96.00 ± 0.30	< 0.1
	500	92.90 ± 0.60	
	250	89.45 ± 0.90	
	125	86.23 ± 0.22	

Result expressed as% inhibition cmean ± SEM of n = 3) and IC_50_ values. Values significantly different as compare to positive control, *:P < 0.05, **:P < 0.01, ***:P < 0.001.

## Discussion

Discovery and development of new antioxidant drugs is among the most exciting areas of pharmacological research. Oxygen is a vital element of aerobic life, but under certain conditions it can seriously influence our health by formation of reactive oxygen species (free radicals) leading to some potentially dangerous diseases, like coronary heart disease, diabetes, atherosclerosis, neurodegenerative disorders (AD & Dementia), cancer, immune-suppression, ageing and ulcer [33,34]. Most common free radicals include hydroxyl, nitric oxide, superoxide & lipid peroxyl, whereas non-free radicals primarily include singlet oxygen and hydrogen peroxide [35]. Nevertheless, approximately all living organisms are protected from free radicals attack by defense system, such as a protective antioxidant system that diminish the rate of free radical formation along with another system which create chain-breaking antioxidants to scavenge & stabilize free radicals. However, when the rate of free radical generation exceeds the capacity of defense mechanisms, extensive tissue injury results [36]. Consequently, drugs with free radical scavenging abilities are useful for the prevention and therapy of these diseases [37]. Antioxidant compounds are known to show their biochemical effects via several mechanisms, including hindrance of chain initiation, chelation of metal ions, breakdown of peroxides, sustained hydrogen abstraction, reductive ability and radical scavenging. Hence, numerous methods are proposed to assess the antioxidant activity. DPPH is an extensively used model system to evaluate the free radical scavenging potential of drugs [38]. DPPH radicals are scavenged by antioxidants through the donation of hydrogen, thus forming reduced DPPH-H, which change the color from purple to yellow following reduction and is quantified by analyzing absorbance at wavelength 517 nm [39]. The ABTS assay is based on the antioxidant capacity of the samples to prevent the oxidation of ABTS to ABTS^++^ radical cation.

**Figure 3:**
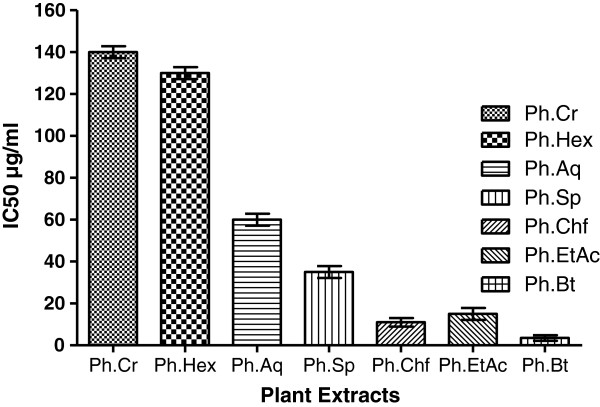
**IC**_**50 **_**values For antioxidant activity of Plant extracts using DPPH assay.**

Phenolics are a class of antioxidant compounds which function as free radical terminators [15]. Previous reports indicate that the free radicals scavenging efficiency of phenolics is dependent on their molecular weight, presence of aromatic rings and nature of OH group’s substitution [40]. Figure 1 shows extraction yield of phenolics (mg GAE/g of sample) indicating that Ph.EtAc, Ph.Chf and, Ph.Bt expressed high concentrations of phenolics. Results of DPPH and ABTS scavenging activities well correlates with phenolic content and might be attributed to presence of high molecular phenolics in addition to flavonoids in these fractions of plant. Median inhibitory values (IC_50_) were 3, 10, 15 and 35 μg/ml for Ph.Bt, Ph.Chf, Ph.EtAc and Ph.Sp fractions in DPPH free radical scavenging assay. Likewise the IC_50_ values for all fractions in ABTS assay were < 0.01 which are comparable to positive control galantamine (IC_50_ < 0.01) Figure 3 and Table 1. Our findings indicate that *P. hydropiper* is enriched with antioxidant compounds and show its possible effectiveness in the management of free radicals induced disorders especially neurodegenerative diseases.

Medicinal plants having therapeutic potential for the treatment of neurodegenerative diseases like AD, Epilepsy and Parkinsonism have been extensively explored, still there is a continuous search for new drugs like galanthamine [24,41]. There are several reports which specify the biological potential of plants as AChE inhibitors *in-vitro* as well as memory enhancers *in-vivo *[41,42]. Table 2 shows AChE inhibitory activity (%) and IC_50_ values of the plant extracts. All fractions showed concentration dependent AChE inhibitory activity. Results of the current study revealed that Ph.Hex was most effective against AChE causing 87.49% enzyme inhibition with IC_50_ 35 μg/ml. Other fractions were also effective at 1 mg/ml concentration. The concentrations of the crude plant extracts that inhibited BChE activity by 50% (IC_50_) are presented in the Table. Our results indicate that *P. hydropiper* extracts are equally effective against BChE. The strongest BChE inhibitory activities were exhibited by Ph.Aq and Ph.Hex fractions, causing 87.62 and 90.30% inhibition with IC_50_ values of 3 and 40 μg/ml respectively.

## Conclusions

In the light of our findings, it can be concluded that most fractions of our plant screened herein exhibited high antioxidant potential and can be related to presence of high molecular weight phenolics. The plant has also showed inhibitory activity against AChE & BChE enzymes in dose-dependent way. This warrant further investigations to exploit the potential use of the bioactive compounds in the treatment of neurodegenerative diseases. Further research linked to the isolation of the bioactive compounds via bioassay-guided isolation is in progress in our laboratory.

## Abbreviations

(Ph.Cr): Crude methanolic extract of *P. hydropiper*; (Ph.Hex): *n*-hexane fraction of *P. hydropiper*; (Ph.Chf): chloroform fraction of *P. hydropiper*; (Ph.EtAc): Ethyl acetate fraction of *P. hydropiper*; (Ph.Bt): *n*-Butanol fraction of *P. hydropiper*; (Ph.Aq): Aqueous fraction of *P. hydropiper*; (Ph.Sp): Saponins fraction of *P. hydropiper*; (AChE): Acetylcholinesterase (BChE), Butyrylcholinesterase; (BHT): Butylated hydroxy toluene; (BHA): Butylated hydroxy anisole; (ACh): Acetylcholine; (AD): Alzheimer's disease; (FCR): Folin Ciocalteu reagent.

## Competing interests

The authors declare that they have no competing interest.

## Authors’ contributions

MA and SA carried out experimental work, data collection and evaluation, literature search and manuscript preparation. MJ and FU supervised research work. JA, AS and MI refined the manuscript for publication. All authors read and approved the final manuscript for publication.

## Pre-publication history

The pre-publication history for this paper can be accessed here:

http://www.biomedcentral.com/1472-6882/14/145/prepub
